# Unique Pattern of Intrahepatic T-cell Clonality in Biliary Atresia Livers Versus Intestinal Controls: A Pilot Study

**DOI:** 10.1097/PG9.0000000000000053

**Published:** 2021-02-24

**Authors:** Sina Ogholikhan, Kathleen B. Schwarz, Robert Anders

**Affiliations:** From the * Division of Pediatric Gastroenterology, Nutrition and Hepatology, The Johns Hopkins University School of Medicine, Baltimore, MD; the † Division of Pathology, The Johns Hopkins University School of Medicine, Baltimore, MD.

**Keywords:** infantile cholangiopathy, immunopathogenesis, unique antigens

## Abstract

Biliary atresia (BA) is a rare infantile cholangiopathy of unclear etiology proposed by some to be due to virus-induced autoreactive T-cell-mediated inflammation. The hallmark of T cell activity is clonal expansion of T lymphocytes expressing similar T-cell receptor (TCR) variable regions of the β-chain.

**Objective:**

To test our hypothesis that BA liver tissues would show clonal expansion of 1 or several TCRs.

**Methods:**

The complementarity-determining region 3 region of the β-chain of the TCR was characterized using next-generation sequencing of 7 BA liver samples (age 51 ± 14 days) and 9 intestinal control samples (age 38 ± 16 days). Following sequencing, clonality scores, various VDJ recombinations, total and productive templates, and complementarity-determining region 3 length were measured using the immunoSEQ Analyzer.

**Results:**

Next-generation sequencing revealed 1 common TCR rearrangement in 3 BA samples not found in controls. There was a highly diverse TCR population among BA liver and the control samples. The clonality scores ranged from 0.0004 to 0.0062 using a Shannon’s entropy score, with numbers close to 0 being highly diverse and numbers close to 1 being highly clonal. The most common TCR VDJ recombinations comprised 1.47–12.9% of the total population of TCR for the BA tissues and 1.05–10.3% for the control samples.

**Conclusions:**

Our results show a highly diverse TCR repertoire among all of our samples. However, predominant TCR clonality was not found in any sample. Further studies are required for any possible antigenic triggers responsible for the unique T-cell rearrangements observed in the BA samples.

## Abstract 2

Supplemental Digital Content is available in the text.

**What Is Known**

- The rotavirus mouse model of BA suggests that T-cells directed against bile ducts may play a role in the inflammatory cholangiopathy but the search for viral or other antigens in human BA tissues has been unproductive.
- The hallmark of T cell activity is clonal expansion of T lymphocytes expressing similar T-cell receptor variable regions of the β-chain.

**What Is New**

- Three BA livers samples had unique T cell receptor sequences.
- Failure to find these sequences in age-related control intestinal tissues suggests unique antigens triggering some BA.

**B**iliary atresia (BA) is a rare disease of unclear etiology, in which obstruction of the biliary tree causes severe cholestasis leading to cirrhosis and ultimate death if left untreated. It accounts for 40–50% of all pediatric liver transplants (1). A widely-accepted theory regarding the etiology of BA is bile duct injury due to virus-induced autoreactive T cell-mediated inflammation or other immune-mediated responses (2).

Mack et al (3) previously reported diminished intrahepatic regulatory T cell function in a rotavirus-induced mouse model of BA. A number of studies have demonstrated abundant T cell infiltrates in the livers of human BA tissue (4,5). Mack et al also that demonstrated that T cells present in the portal tracts and extrahepatic bile duct remnant of BA patients are composed of oligoclonally expanded T-cell populations, suggesting their accumulation in response to conventional antigenic stimulation. However, no study has looked at the specific T-cell receptor (TCR) receptor profiles using next-generation sequencing (NGS).

A hallmark of T cell activity, whether in an autoimmune disease or a viral infection, is clonal expansion of specific T lymphocytes. T cells use cell surface alpha/beta (or less frequently gamma/delta) receptors to engage epitopes presented in major histocompatibility molecules. Functional TCR α-chain and β-chain genes result from somatic rearrangement of the variable VDJ region gene segments in the complementarity-determining region 3 (CDR3) region of the TCR. This process makes the potential TCR repertoire enormous with greater than 10^7^ sequence possibilities (6). When a particular epitope stimulates a T cell receptor, the T cell proliferates. T cell proliferation is measured in a T cell population by clonal expansion. A single T cell clone expands and becomes a greater proportion of the total T lymphocytes, therefore demonstrating clonality. The purpose of this study was to investigate our hypothesis that TCRs (as characterized by NGS) in BA liver tissues will show clonal expansion of 1 or several TCRs when compared to a control group.

## METHODS

### Study Population

Between 2011 and 2016, wedge liver biopsies were collected from 7 patients with isolated BA at the time of the Kasai portoenterostomy and were formalin-fixed and embedded in paraffin (FFPE). Intestinal tissue was obtained from 9 age-matched infants with isolated intestinal anatomical defects. FFPE intestinal samples were selected for the control patients given the lack of healthy neonatal liver samples. Patient characteristics from the BA infants are listed in Supplemental Table 1; http://links.lww.com/PG9/A20. Exclusion criteria included known bacterial or viral infection at the time of surgery. All patients, both controls and BA, were below the age of 14 weeks. This study was approved by The Johns Hopkins Hospital Institutional Review Board.

### Tissue Collection and Extraction

The Johns Hopkins pathology database from 2013 to 2016 was searched for patients and controls. Nine BA cases were identified. However, 2 were excluded due to other anatomical defects; none of the others had extrahepatic congenital anomalies. For the BA patients, liver wedge biopsies that had been obtained at the time of the Kasai surgery were used. The TCR technique requires 3 μm × 9 samples of tissue. This tissue was formalin-fixed paraffin-embedded and was requested through the Department of Pathology at Johns Hopkins University. Hematoxylin and eosin stained histology were reviewed for confirmation of BA and block sections were confirmed.

Due to the lack of healthy infant liver samples, intestinal tissue was seen as a comparable tissue choice for normal T-cell identification. Using the same pathology database, cases were identified from 2012 to 2016. These cases included isolated intestinal atresias with no other anatomical or systemic disorders. Chromosomal abnormalities such as trisomy 21 were excluded along with any identified viral or bacterial infection (confirmed using blood, urine, and viral culture). Nine cases were identified and the hematoxylin and eosin slides confirmed healthy margins. These blocks were selected and cut into 3 μm sections.

### T-cell Receptor Next-generation Sequencing

The rearranged TCRβ CDR3 was amplified from gDNA using a multiplex polymerase chain reaction and sequenced by Adaptive Biotechnologies (Seattle, WA) using their ImmunoSEQ assay. Intestinal and liver TCRβ repertoires were sequenced at survey resolution according to company’s guidelines for samples. Polymerase chain reaction and sequencing errors were removed, and for each unique sequence, the nucleotide and predicted amino acid sequence, V (variable), D (diversity), and J (joining) genes and the number of sequencing reads were determined. The data were further sorted to exclude any sequence with an out-of-frame rearrangement or a stop codon in the complementarity-determining region 3, and the frequency was determined for each of the remaining productive unique sequences (nucleotide clonotypes).

Data were analyzed using the ImmunoSEQ Analyzer with general bioinformatics strategies. T cell clonality was calculated as 1 − (Shannon’s Entropy)/log_2_(number of nucleotide clonotypes). A clonality score of 1 indicates a monoclonal sample, whereas a score of 0 indicates a maximally diverse sample.

## RESULTS

The immunoSEQ analyzing tool provided the following TCR data: the total templates, productive templates, total and productive rearrangements, clonality scores, and the maximum productive frequency of the TCRs. Productivity is the count of unique rearrangements in the sample that are in-frame, do not contain a stop codon, and can produce a functional protein receptor. The initial sample overview is shown in Table 1, for the BA tissues and Supplemental Table 2; http://links.lww.com/PG9/A21, for the control samples. Our results showed there were 23,520 total templates among all of our 16 tissue samples. Clonality scores were given to each sample ranging from 0 to 1. Values near 1 represent samples with one or a few predominant rearrangements (monoclonal or oligoclonal samples) dominating the observed repertoire. Clonality values near 0 represent more polyclonal samples. For our 16 samples, the clonality ranges were from 0.0004 to 0.0062. We then calculated the frequency of a specific productive rearrangement among all productive rearrangements within a sample. This was calculated as the templates for a specific rearrangement divided by the sum of productive templates for a sample.

**TABLE 1.** Summary of TCR Repertoires in BA Patients

Sample name	Total templates*	Total productive templates†	Productive rearrangements‡	Productive clonality§	Max productive frequency (%)¶
BA-1553996	4298	3049	2903	0.0032	0.295179
BA-1429579	575	413	399	0.0023	0.726392
BA-132030	1078	749	690	0.0062	0.801068
BA-1477888	1235	870	850	0.002	0.574713
BA-1418804	1045	658	653	0.0004	0.303951
BA-1453785	170	93	91	0.0018	2.150538
BA-153205	2428	1745	1714	0.0009	0.114613

*Total templates are the sum of templates for all rearrangements in the sample.

†Total productive templates are the sum of templates for all Productive Rearrangements in the sample.

‡Productive rearrangements are the count of unique rearrangements in the sample that are in-frame and do not contain a stop codon. Productive rearrangements can produce a functional protein receptor.

§Productive clonality measure for the sample is calculated over all Productive Rearrangements. Values for clonality range from 0 to 1. Values near 1 represent samples with one or a few predominant rearrangements (monoclonal or oligoclonal samples) dominating the observed repertoire. Clonality values near 0 represent more polyclonal samples. Productive Clonality is calculated by normalizing Productive Entropy using the total number of unique Productive Rearrangements and subtracting the result from 1.

¶Maximum Productive Frequency is the frequency of a specific productive rearrangement among all Productive Rearrangements within a sample. Calculated as the templates for a specific rearrangement divided by the sum of Productive Templates for a sample.

BA = biliary atresia; TCR = T-cell receptor.

The top 10 most common rearrangements comprised 1.47–12.9% of the total population for the BA samples and 1.05–10.3% for the control samples.

### Clonality Score

**FIGURE 1.**
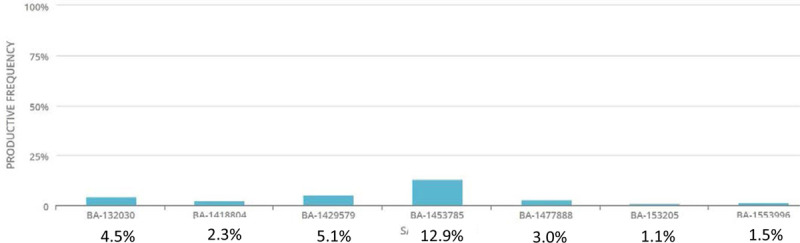
Top 10 productive TCR rearrangements in BA liver. BA = biliary atresia; TCR = T-cell receptor.

**FIGURE 2.**
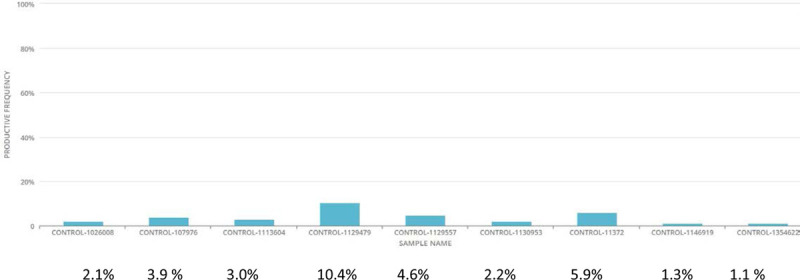
Top 10 productive TCR rearrangements in control tissues. TCR, T-cell receptor.

The clonality scores are given in Table 1, demonstrating a highly diverse TCR population. The figures show the top 10 productive rearrangements in comparison to the entire TCR population for BA (Fig. 1) and intestinal controls (Fig. 2). It is important to note that the 2 highest percentages from the 16 samples (1 BA and 1 control) were the 2 samples with 2 lowest total number of templates. Due to the low number of templates, the top 10 rearrangements represented a much higher percentage of the total population.

In 3 of the 7 BA samples, there was 1 common TCR rearrangement which was not found in any of the intestinal control samples. Two of these 3 patients also had another unique TCR not seen in any of the controls. These unique sequences are shown in Table 2.

**TABLE 2.** Unique TCR Rearrangements in BA Patients

Rearrangement	Present in BA #/%		
…jTACTTCTGTGCCAGCAGTGGGACTAGCGGGAGCACAGATACGCAGTATTTTGGCCCA	BA 132030 0.133511%	BA 1418804 0.0303951%	BA 1553996 0.131191%
…jTACTTCTGTGCCAGCAGTGACTCTAGCGGGAGCACAGATACGCAGTATTTTGCCCA	BA 132030 0.267023%	n/a	BA 1553996 0.29179%

Not present in BA 1429579, BA 1453785, and BA 1477888.

BA = biliary atresia; TCR = T-cell receptor.

## DISCUSSION

Our pilot study demonstrated that we were able to identify TCR repertoires using NGS to study liver and intestinal FFPE biopsies. Both the control group and the BA group showed similar clonality scores ranging from 0.0004 to 0.0062. However, the extremely interesting finding was the 1 common rearrangement seen in 3 of the 7 BA samples, but none of the controls. Two BA patients also had a second unique TCR sequence. This study demonstrated a highly polyclonal TCR repertoire in both BA and control patients using clonality scores. Among single TCR clones and the top 10 clones, both of the BA and control populations demonstrated diverse TCR populations.

NGS of TCRs had not been performed in neonatal tissues previously and the feasibility of this technique demonstrated by our studies promises to have wide applicability not only for BA but for other antigen-driven diseases of neonates. Our data demonstrated that both the BA and control populations had a highly diverse and polyclonal repertoire. There was no single clone that represented more than 2.2% of the entire population and this was a higher percentage due to the low amount of productive templates.

As previously stated, it has been proposed that the pathogenesis of BA involves a viral-induced, progressive autoimmune-mediated injury of bile duct epithelia. An unknown perinatal infection with affinity for the bile duct epithelia could cause initial bile duct epithelial injury. This injury elicits an inflammatory response, even with viral clearance. There is continuous inflammation and injury to the bile duct epithelia through expression of either altered or previously sequestered “self”-antigens. These antigens are now recognized as foreign, eliciting autoreactive T-cell-mediated inflammation. Alternatively, viral proteins may be structurally similar to bile duct epithelial proteins and autoimmunity could be elicited based on the molecular mimicry pathway. Mack et al (7) have shown evidence of this autoimmune theory for both cellular and humoral autoimmunity in a rotavirus-induced murine model of BA. There are many oligoclonal T-cell expansions found in antigen-driven immune responses. These including autoimmune diseases, infections, and malignancies (8). Using this well-established pathogenesis of auto-immune diseases, we set out to determine if the liver-specific T cells in BA showed clonal expansion as a result of a viral-specific antigen response and did find some unique patterns in BA suggesting that the TCR’s were responding to unique antigens.

Characterization of the normal T-cell repertoire of the immature immune system proved to be challenging given that, as expected there were no liver biopsy wedge samples in our pathology database from either healthy age-matched infants or liver disease controls. It was essential to find a tissue capable of demonstrating the T-cell repertoire in a normal immune system. We decided that healthy intestinal margins, obtained in the process of surgical removal of strictured sections of intestine, in infants with isolated intestinal atresia provided a logical set of age-matched control tissues against which to compare the BA samples. By excluding intestinal atresias associated with syndromic disorders, such as Trisomy 21, and by excluding intestinal tissue with any evidence of neonatal necrotizing enterocolitis, we could find age-matched intestinal tissue specimens, while isolate the healthy margins.

Specific rearrangements may potentially be used as a screening tool, especially if corroboration can be found between liver and serum samples. This rearrangement will be investigated further, by perhaps locating its triggering source.

Potential limitations to this study include the lack of true healthy age-matched liver controls as mentioned above as well as the lack of age-matched liver controls obtained from patients with neonatal liver disease other than BA and the lack of biliary remnants from the BA patients. It is also unclear whether the low amount of templates in a specific sample represented a low number of overall T-cells or whether it was due to poor tissue integrity.

Using NGS, many T cell-targeted therapies have been developed for specific treatments for a number of diseases. Poschke et al conducted a phase I clinical trial of combining dendritic cell vaccination with adoptive T cell transfer in patients with stage IV melanoma using NGS TCR sequencing (9). In another study, Emerson et al utilized sequencing of TCR’s to demonstrate a homogeneous repertoire of tumor-infiltrating lymphocytes in ovarian cancer (10).

In addition, Emerson et al were able to use specific TCRβ sequences fin CMV positive patients identified by serostatus. These specific TCR sequences proved to be highly specific and sensitive, diagnosing CMV status in patients whose CMV serostatus was unknown (11). Similarly to this study, if additional BA liver samples show the unique TCR rearrangement as we found in our 3 BA liver samples, this could perhaps be applied to a screening tool for BA in addition to or possibly in replacement of more invasive diagnosis techniques. Further studies are required to identify the triggering factor in the common rearrangement found among the 3 BA samples. If a trigger is found, this could lead to a possible treatment of the initial insult.

## CONCLUSIONS

Our data have demonstrated the feasibility of characterizing the TCR repertoire in FFPE tissue samples obtained from infants, a technique which is likely to have wide applicability in the future for understanding the immunopathogenesis of inflammatory diseases in this age group. Using NGS, our study demonstrated 2 specific TCR rearrangements found only in BA patients. Three of the 7 BA patients had 1 common TCR rearrangement and 2 of the 3 had a second unique sequence that differed from the first by only 1 nucleotide. Further studies are required for any possible antigenic triggers responsible for the unique T-cell rearrangements observed in the BA samples.

## Supplementary Material


