# Supplementary material for: Unique Pattern of Intrahepatic T-cell Clonality in Biliary Atresia Livers Versus Intestinal Controls: A Pilot Study
Source: JPGN Rep. 2021 Feb 24;2(2):e053. doi: 10.1097/PG9.0000000000000053 (PMC10191516; doi:10.1097/PG9.0000000000000053)
Supplement: Supplementary file 1 [file pg9-2-e053-s001.pdf]

Supplemental Table 1.

Demographic and laboratory features of BA patients

Sex	4M 3F	Reference range
Age at Kasai (days) (mean/median)	24 – 95<br>(51/42)	
Aspartate aminotransferase IU/L	107 - 331	12 - 47
Alanine aminotransferase IU/L	53 – 322	14 - 54
Total bilirubin mg/dl	3.1 – 11.6	0.3 - 1.8
Direct bilirubin mg/dl	2.5 – 8.6	0.1 – 0.6
Alkaline phosphatase IU/L	156 – 624	52 - 222
Albumin g/dl	1.9 – 4.1	3.4 - 5.1
White blood cells/cc ^{3}	4.2 – 15.9	4.5 - 13.5
Hemoglobin g/dl	8.9 – 16.1	11.3 – 16.0
Platelets x 10 ^{6} cc ^{3}	182 – 686	150 - 450
Prothrombin time (sec)	10.3 – 12.5	9.0 – 11.5
International Normalized Ratio	1.0 – 1.2	0.9 – 1.1
Gamma Glutamyl transferase (U/L)	82 - 1010	3.0 - 22
Liver biopsy CD3 T cells density (cells/tissue area um^2)	4.25E-04 (mean)<br>3.29E-04 (median)	Range<br>2.32E-04 - 8.25E-04
