# Supplementary material for: Unique Pattern of Intrahepatic T-cell Clonality in Biliary Atresia Livers Versus Intestinal Controls: A Pilot Study
Source: JPGN Rep. 2021 Feb 24;2(2):e053. doi: 10.1097/PG9.0000000000000053 (PMC10191516; doi:10.1097/PG9.0000000000000053)
Supplement: Supplementary file 2 [file pg9-2-e053-s002.pdf]

Supplemental Table 2. TCR Repertoires in Control Infants

Sample name	Total templates (A)	Total Productive Templates (B)	Productive Rearrangements (C)	Productive Clonality (D)	Max Productive Frequency (E)
Control-1130953	2,046	1,385	1,336	0.0024	0.288809%
Control - 1129479	203	135	131	0.0031	2.222222%
Control - 1129557	648	432	418	0.002	0.462963%
Control - 1026008	1,435	1,036	1,025	0.0007	0.289575%
Control - 1146919	2,290	1,728	1,716	0.0005	0.231481%
Control - 1354622	4,389	3,125	3,031	0.0017	0.128%
Control - 107976	507	328	325	0.0006	0.609756%
Control - 11372	559	352	341	0.0022	0.852273%
Control - 1113604	614	390	388	0.0006	0.7609231%

A. Total templates are the sum of templates for all rearrangements in the sample.
B. Total productive templates are the sum of templates for all Productive Rearrangements in the sample.
C. Productive rearrangements are the count of unique rearrangements in the sample that are in-frame and do not contain a stop codon. Productive rearrangements can produce a functional protein receptor.
D. Productive clonality measure for the sample is calculated over all Productive Rearrangements. Values for clonality range from 0 to 1. Values near 1 represent samples

with one or a few predominant rearrangements (monoclonal or oligoclonal samples) dominating the observed repertoire. Clonality values near 0 represent more polyclonal samples. Productive Clonality is calculated by normalizing Productive Entropy using the total number of unique Productive Rearrangements and subtracting the result from 1.

E. Maximum Productive Frequency is the frequency of a specific productive rearrangement among all Productive Rearrangements within a sample. Calculated as the templates for a specific rearrangement divided by the sum of Productive Templates for a sample.
